# Temperature Effects on Biotic and Abiotic Mechanisms of Nitrogen Removal Enhanced by Polybutylene Succinate in Synthetic Low-Carbon Wastewater

**DOI:** 10.3390/microorganisms14102167

**Published:** 2026-09-25

**Authors:** Jia Liu, Heng Wu, Xiaojun Guo, Jianhua Yuan

**Affiliations:** 1 College of Mechanical and Electrical Engineering, Tarim University, Alar 843300, China; 120200011@taru.edu.cn (J.L.); 120210036@taru.edu.cn (X.G.); 2 China-Croatia Belt and Road Joint Laboratory on Biodiversity and Ecosystem Services, Agricultural Microbial Agents Key Laboratory of Sichuan Province, Chengdu Institute of Biology, Chinese Academy of Sciences, Chengdu 610213, China; 3 Hunan Province Key Laboratory of Typical Environmental Pollution and Health Hazards, School of Public Health, Hengyang Medical School, University of South China, Hengyang 421001, China

**Keywords:** polybutylene succinate, low-carbon wastewater, microbial community, metabolic mechanisms, molecular diffusion, machine learning

## Abstract

Using polybutylene succinate (PBS) to enhance nitrogen removal from low-carbon wastewater has gained increasing attention, yet its temperature adaptability remains unclear. PBS-enhanced nitrogen removal and its coupled bio-abiotic mechanisms were evaluated from 5 to 34 °C in a lab-scale system treating synthetic wastewater. High total nitrogen (TN) removal efficiency of 89.9% was achieved at 23–25 °C with influent TN of 60.8 mg/L, indicating an optimal temperature window. Mechanistic analysis revealed that temperatures above 23 °C promoted higher microbial community diversity, fostering a consortium dominated by *unclassified_Burkholderiaceae* (7.73–13.93%) and *Leucobacter* (6.29–7.47%), accompanied by enrichment of carbon/nitrogen metabolism-related genes and efficient nitrogen removal. Temperatures above 23 °C also maintained a high organic matter diffusion coefficient (0.043–0.222 Å^2^/ps), enabling microorganisms to fully utilize carbon sources. A random forest model linked the diffusion coefficient closely to pollutant removal efficiency and identified low-temperature-adapted genera (*Flavobacterium*, *Dechloromonas*) and functional genes (*gapdh*, *fba*, *gpmI*, *napA*, *napB*, *nosZ*), offering insights for enhancing low-temperature nitrogen removal. The model predicted effluent TN well (R^2^ > 0.98) and confirmed effluent NH_4_^+^-N as the dominant driver of TN variation. Pilot-scale tests with real wastewater are still needed. This study provides theoretical support for PBS-enhanced nitrogen removal in low-carbon wastewater.

## 1. Introduction

Excessive nitrogen leads to water eutrophication, making nitrogen pollution one of the most concerning environmental issues worldwide [1,2]. Nitrogen in wastewater can be removed through microbial metabolism; that is, ammonia nitrogen (NH_4_^+^-N) is converted to nitrate (NO_3_^−^-N) via aerobic nitrification, and then transformed into nitrogen gas through heterotrophic denitrification [3]. However, heterotrophic denitrification relies on organic matter as an electron donor [4]. In low-carbon wastewater, the lack of sufficient electron donors results in reduced nitrogen removal efficiency [5,6].

The addition of external carbon sources is commonly adopted to enhance low-carbon wastewater treatment [7]. Solid carbon sources have attracted widespread attention owing to their unique advantages, which include serving as attachment carriers for microorganisms to promote microbial enrichment and providing electron donors to facilitate microbial metabolism [8]. Based on their physicochemical properties, solid carbon sources can be divided into synthetic carbon sources and natural carbon sources [9]. Alitaleshi et al. investigated the nitrogen removal enhancement performance of wheat husks on low-carbon wastewater and reported that 86% of NH_4_^+^-N was removed at an influent NH_4_^+^-N concentration of 7 mg/L [10]. Additionally, corn cobs used as a carbon source showed even better performance, achieving a 97% removal efficiency for influent NO_3_^−^-N at 50 mg/L [11]. Although these natural carbon sources can improve nitrogen removal efficiency, they contain large amounts of impurities that cannot be fully utilized, resulting in increased excess sludge production and a risk of secondary pollution [12]. Moreover, their uneven texture causes unstable carbon release, which is unfavorable for supplying electron donors to microorganisms and achieving stable nitrogen removal [13].

In contrast, synthetic carbon sources are cleaner because they can be completely degraded without accumulating secondary pollutants and can deliver a steady supply of organic carbon [14]. As a representative synthetic carbon source, polybutylene succinate (PBS) has been widely applied to enhance nitrogen removal in low-carbon wastewater treatment [14,15]. Its technical feasibility and superior performance over conventional carbon sources (e.g., sodium acetate) have been fully verified in previous studies [14,15]. Current research still exhibits obvious limitations and fails to establish a systematic and comprehensive theoretical basis for practical engineering applications. The main limitations are summarized as follows: (1) Existing studies predominantly focus on the effects of conventional operational parameters, such as hydraulic retention time (HRT) and dissolved oxygen (DO), on the nitrogen removal performance of PBS-enhanced systems [5,8]. However, the adaptability of the PBS-based nitrogen removal process over a wide temperature range remains underexplored, resulting in insufficient theoretical support for its practical engineering promotion. For instance, the average annual water temperature in northern China is below 10 °C, while the year-round water temperature in southern China stays above 20 °C [16,17]. (2) Previous studies have extensively investigated the responses of PBS-enriched microbial communities to conventional operational parameters including HRT and DO [8,18], yet the successional characteristics and intrinsic driving mechanisms of temperature-dependent functional microbiota remain largely unclear. This knowledge gap hinders our understanding of the microbial regulatory mechanisms governing nitrogen removal under variable temperature conditions. (3) The critical role of temperature-driven molecular mass transfer processes in assisting and improving nitrogen removal efficiency has been neglected in prior research [1,4], leading to an incomplete theoretical framework for PBS-mediated nitrogen removal from low-carbon wastewater. (4) More importantly, compared with conventional activated sludge models, machine learning approaches can more efficiently and accurately identify the key limiting factors for nitrogen removal efficiency by integrating biotic and abiotic variables, which contributes to the precise optimization of practical operational parameters [19]. However, research integrating machine learning algorithms with PBS-based low-carbon wastewater treatment is still lacking, which restricts parameter optimization and large-scale application of this process.

Based on the above discussion, this study aims to: (1) evaluate the enhancement performance of the PBS system for wastewater treatment across a temperature range of 5–34 °C to determine its practical application scope; (2) reveal the characteristics of temperature-driven microbial community remodeling and carbon/nitrogen metabolism-related gene enrichment to clarify the key microbial mechanisms; (3) decipher the features of temperature-mediated molecular mass transfer processes to supplement the underlying abiotic mechanisms; (4) identify the key driving and limiting factors for nitrogen removal using a random forest-based machine learning model to support process optimization. This work provides solid theoretical guidance for the design and optimization of temperature control strategies in low-carbon wastewater treatment.

## 2. Materials and Methods

### 2.1. Experimental Setup

A continuous flow mode was adopted in the experiment, with four beakers operated simultaneously at temperature gradients of 5–7, 14–16, 23–25, and 32–34 °C (Figure S1). Each beaker had an effective working volume of 0.8 L and was placed in a thermostatic water bath to maintain a stable temperature, with real-time temperature monitoring implemented throughout the experiment to allow timely adjustments. Biofilm-attached PBS was sampled from a steadily operating biofilm reactor, and then placed in the beakers as carriers at a dosage ratio of 30% (*v*/*v*). An aeration diffuser was installed in each beaker to supply oxygen and maintain the target DO level at approximately 3 mg/L.

### 2.2. Carriers and Wastewater Characteristics

The PBS used in this study had a relative molecular weight of 80,000, a density of 1.146 kg/L, and a diameter of approximately 3 mm, and was purchased from eSUN New Materials Co., Ltd. (Shenzhen, China). The synthetic wastewater was characterized by a chemical oxygen demand (COD) concentration ranging from 190 to 217 mg/L, an NH_4_^+^-N concentration from 45.9 to 51.5 mg/L, and a NO_3_^−^-N concentration from 10.1 to 13.0 mg/L. The synthetic wastewater was prepared by dissolving C_6_H_12_O_6_, KNO_3_, NH_4_Cl, and trace element solution in tap water. The trace nutrient solution contained the following components: MgSO_4_·7H_2_O (2 g/L), MnSO_4_·H_2_O (0.1 g/L), CaCl_2_ (1.5 g/L), and FeSO_4_·7H_2_O (0.1 g/L). The trace element solution was added at a ratio of 5% (*v*/*v*).

### 2.3. Experimental Procedures

Activated sludge with a total suspended solids (TSS) concentration of 6.5 g/L was used for biofilm colonization on PBS carriers. After biofilm formation, the carriers were added to these beakers in sequence at a ratio of 30% (*v*/*v*). All beaker experiments were conducted in a continuous flow mode, with the operating temperatures set to 5–7, 14–16, 23–25, and 32–34 °C, respectively. Influent was delivered to each beaker using a micro-peristaltic pump, while effluent was withdrawn from the top of each beaker via another micro-peristaltic pump. Based on the previous literature, the HRT was set at 18 h [20], and the DO concentration was controlled at approximately 3 mg/L [8]. The treatment efficiency was evaluated daily by measuring the concentrations of COD, NH_4_^+^-N, NO_3_^−^-N, and total nitrogen (TN) in the effluent. After 24 consecutive days of operation, the biofilms attached to the PBS carriers were collected and stored at −80 °C for subsequent microbial analysis.

Three replicate beaker reactors were operated for each temperature condition to obtain independent water-quality data, from which the mean values and standard deviations were calculated. For high-throughput sequencing, biofilm biomass from the three parallel reactors at the same temperature was pooled in equal proportions to improve sample representativeness and reduce random experimental error.

### 2.4. DNA Extraction, Polymerase Chain Reaction and 16S High-Throughput Sequencing

For microbial community analysis during the experiment, biofilm samples were collected from PBS carriers in beakers maintained at different temperatures (5–7, 14–16, 23–25, and 32–34 °C), before being subjected to Illumina MiSeq sequencing. Total microbial genomic DNA was isolated from these biosamples using the E.Z.N.A.^®^ Soil DNA Kit (Omega Bio-tek, Norcross, GA, USA), strictly following the manufacturer’s recommended protocols. After DNA extraction, DNA quality and concentration were assessed using 1.0% agarose gel electrophoresis and a NanoDrop 2000 spectrophotometer (Thermo Scientific, Wilmington, DE, USA), and the qualified DNA samples were preserved at −80 °C for later experimental use. To amplify the hypervariable V3-V4 region of the bacterial 16S rRNA gene, primer pairs 338F (5′-ACTCCTACGGGAGGCAGCAG-3′) and 806R (5′-GGACTACHVGGGTWTCTAAT-3′) were used, with a T100 Thermal Cycler (BIO-RAD, Hercules, CA, USA) serving as the amplification instrument. The PCR reaction system was prepared as follows: 4 μL of 5× Fast Pfu buffer, 2 μL of 2.5 mM dNTPs, 0.8 μL of each primer (5 μM), 0.4 μL of Fast Pfu polymerase, 10 ng of template DNA, and ddH_2_O added to bring the total volume to 20 μL. The PCR amplification procedure included the following steps: an initial denaturation step at 95 °C for 3 min; 27 cycles consisting of denaturation at 95 °C for 30 s, annealing at 55 °C for 30 s, and extension at 72 °C for 45 s; a final extension phase at 72 °C for 10 min; and a holding step at 4 °C to terminate the reaction. After amplification, PCR products were recovered from 2% agarose gels, purified using the PCR Clean-Up Kit (YuHua, Shanghai, China) in accordance with the manufacturer’s instructions, and quantified using a Qubit 4.0 fluorometer (Thermo Fisher Scientific, Waltham, MA, USA). Purified amplicons were mixed in equimolar proportions and then sent for paired-end sequencing on an Illumina PE300 platform (Illumina, San Diego, CA, USA). The sequencing process was carried out by Majorbio Bio-Pharm Technology Co., Ltd. (Shanghai, China) following standard operating procedures. For rigorous data quality control, the integrity of barcodes and adapters was checked first; sequences shorter than 50 bp were removed, and bases with a quality score below 20 were filtered out. Finally, paired-end reads were merged under the following parameters: a minimum overlap length of 10 bp and a maximum mismatch ratio of 0.2 allowed.

### 2.5. Analytical Methods for Microbial Data and Physicochemical Indicators

Chao, Shannon, Sob, Shannoneven, and Coverage indices were calculated using Mothur v1.30.1 [21]. To explore inter-genus interaction patterns, a microbial co-occurrence network was constructed based on genus-level community profiles [22]. To predict the potential functional gene abundances associated with nitrogen and carbon metabolic pathways in the biological samples, the Phylogenetic Investigation of Communities by Reconstruction of Unobserved States 2 tool was used with reference to the Kyoto Encyclopedia of Genes and Genomes (KEGG) database [23]. In the wastewater, NH_4_^+^-N, NO_3_^−^-N, TN, and COD were determined using the Nessler reagent spectrophotometric method, nitrate nitrogen ultraviolet spectrophotometry, alkaline potassium persulfate digestion-ultraviolet spectrophotometry, and potassium dichromate spectrophotometric method, respectively [24]. TSS were measured via the gravimetric method [24].

### 2.6. Molecular Simulation Method

Details of the amorphous unit model construction were as follows: The simulation was performed using the Forcite module in Materials Studio 2020 software. Molecules used for constructing the amorphous unit model included NH_4_^+^-N, H_2_O, C_6_H_12_O_6_, and NO_3_^−^-N molecules. The COMPASS III force field was employed for charge assignment: the net charge of regular molecules was zero, while NH_4_^+^-N molecules (calculated as NH_4_^+^) carried one positive charge and NO_3_^−^-N molecules (calculated as NO_3_^−^) carried one negative charge. The amorphous unit was built based on the average concentration of each substance after the wastewater treatment system reached a stable state. The density was set to 1.0 g/cm^3^, and the simulation was configured to output 10 frames.

Details of geometry optimization, molecular dynamics simulation, and diffusion coefficient calculation were as follows: The optimal frame was selected from the amorphous unit for geometry optimization, with the force field parameters remaining unchanged. The maximum number of iterations was set to 5000. After completion, if the “Normal” status was displayed, it indicated that annealing was unnecessary. Subsequently, the optimized frame was selected for molecular dynamics simulation. The simulation was conducted under the isothermal-isobaric ensemble (NPT ensemble), with temperatures set to 279.15 K, 288.15 K, 297.15 K, and 306.15 K corresponding to 6 °C, 15 °C, 24 °C, and 33 °C, respectively. The simulation time step was 1 fs, the total simulation time was 300 ps, and data were output every 10,000 steps (per frame). Based on the results of molecular dynamics simulation, various small-molecule configurations were selected from the amorphous unit. The stable period between 10 ps and 300 ps was chosen to analyze the mean square displacement and calculate the diffusion coefficient.

Details of charge analysis were as follows: Charge analysis was performed using Materials Studio 2020 software. Three representative molecular models were constructed: NH_4_^+^-N, C_6_H_12_O_6_, and NO_3_^−^-N. Since an implicit-solvent model was adopted for data analysis, explicit water molecules were not included in the simulation cell. An amorphous unit cell was constructed using these three molecules based on their concentrations, followed by geometry optimization of the unit cell. The CASTEP module and charge density difference method were used separately for the analysis.

### 2.7. Machine Learning Method

The random forest model was selected for machine learning. Based on individual decision trees as the base model, random forest features a clear and interpretable decision-making process and does not overly rely on data distribution, making it highly suitable for handling practical data with complex distributions [25]. The combination of multiple decision trees can effectively reduce the model error rate, mitigate the risks of overfitting and underfitting, and improve generalization ability. The machine learning model was built using Python 3.7, with the modeling process including steps such as data preprocessing, model training with hyperparameter optimization, and model evaluation.

Data features were as follows: The dataset was derived from gene data, genus data, diffusion coefficient, and pollutant concentrations (COD, TN, NO_3_^−^-N, NH_4_^+^-N). The SMOGN algorithm was applied to augment the pollutant concentration data, yielding a final dataset of 200 samples. The resulting dataset was then split into a training set and a test set at a ratio of 4:1 for subsequent prediction and model fitting. Model training and hyperparameter optimization were both conducted on the training set. The TN concentration in the effluent was selected as the prediction target, while the input features included the NH_4_^+^-N concentration, COD concentration, C_6_H_12_O_6_ diffusion coefficient, H_2_O diffusion coefficient, NH_4_^+^-N diffusion coefficient, and NO_3_^−^-N diffusion coefficient in the effluent.

Details of hyperparameter optimization were as follows: GridSearchCV was used for model hyperparameter tuning. The main hyperparameters optimized and their ranges were as follows: n_estimators [100, 200, 300], max_depth [5, 10, 15], min_samples_split [2, 4, 6], and min_samples_leaf [1, 2, 3]. Adjusting parameters such as max_depth, min_samples_split, and min_samples_leaf can control the complexity of decision trees, preventing model overfitting or underfitting. Adjusting n_estimators can balance model performance and computation time.

Details of model performance evaluation were as follows: The trained and hyperparameter-optimized model was used for prediction on the test set to evaluate its accuracy. Since a regression prediction model for continuous variables was constructed, R^2^, root mean square error (RMSE), and mean square error (MSE) were selected to assess the model’s prediction performance and accuracy.

### 2.8. Statistical Analysis

Measurements of pollutant concentrations were performed in triplicate. The mean, standard deviation, and significance (*t*-test) were determined in Origin 2021. Significance levels were set at ** *p* < 0.01 and * *p* < 0.05.

## 3. Results and Discussions

### 3.1. Reducing Temperature Leads to a Decrease in the Nitrogen Removal Efficiency

The COD, NH_4_^+^-N, NO_3_^−^-N, and TN removal efficiencies of the process were continuously monitored over a 24-day operation period. As shown in Figure 1a, at 32–34 °C, the effluent COD concentration decreased gradually from 147 mg/L on the first day, and after stabilization (the last 5 days), the effluent concentration was only 22 mg/L, which met the discharge requirements of wastewater treatment plants specified in China (<50 mg/L). Meanwhile, the NH_4_^+^-N concentration gradually stabilized from 15.4 mg/L on the first day to 1 mg/L on the 24th day, far below the discharge threshold (5 mg/L) of wastewater treatment plants (WWTPs) (Figure 1b). As the main nitrogen component in WWTPs tailwater, the effluent NO_3_^−^-N concentration was also monitored to calculate its removal efficiency (Figure 1c). The stabilized effluent concentration of NO_3_^−^-N was only 2.7 mg/L, confirming the high efficiency of the denitrification process. As a key indicator, TN directly reflected whether the effluent met discharge standards. The average influent TN concentration was 60.8 mg/L, while the stabilized effluent concentration was only 5.4 mg/L (below the threshold of 15 mg/L) (Figure 1d).

In addition, at 23–25 °C, the stabilized effluent COD concentration was only 24 mg/L, with an average removal efficiency of up to 88.2%, which was similar to the 89.2% at 32–34 °C. The stabilized effluent NH_4_^+^-N concentration was 2.8 mg/L, also below the WWTPs’ discharge threshold. The stabilized effluent concentration of NO_3_^−^-N was only 1.9 mg/L, and its removal efficiency further increased from 76.4% at the 32–34 °C to 83.4%, indicating that 23–25 °C was more suitable for the heterotrophic denitrification process. The stabilized effluent TN concentration was 6.1 mg/L, with an average removal efficiency of up to 89.9%. Operation at 23–34 °C effectively removed nitrogen and organic pollutants, and met the discharge requirements of WWTPs.

However, at 14–16 °C, the wastewater treatment performance showed a significantly downward trend. Although the stabilized effluent COD concentration increased to 45 mg/L (still below the WWTPs’ discharge threshold of 50 mg/L), the average removal efficiency decreased to 77.8%, posing a risk of exceeding the standard. The stabilized effluent NH_4_^+^-N concentration increased significantly to 27.3 mg/L, accompanied by a reduced removal efficiency as low as 41.9%, which exceeded the WWTPs’ discharge threshold. Reduced temperature not only inhibited the metabolic activity of heterotrophic microorganisms but also restricted the enrichment of autotrophic nitrifying microorganisms [26,27]. The stabilized concentration of NO_3_^−^-N increased slightly compared with the high-temperature conditions, reaching 2.1 mg/L. The low utilization efficiency of organic matter may be one of the reasons for the weakened denitrification. The weakened microbial activity led to an increase in the stabilized effluent TN concentration to 31.9 mg/L, which obviously also significantly exceeded the WWTPs’ discharge requirements, making it impossible to achieve compliant wastewater discharge.

At 5–7 °C, the wastewater treatment performance further decreased. The stabilized effluent COD concentration was as high as 80.4 mg/L, with a removal efficiency of only 60.3%. The metabolic activity of microorganisms was further inhibited by low temperatures, resulting in a significant decrease in the utilization efficiency of organic matter and thus exceeding the COD discharge standard. Although reduced temperature did not significantly alter the peak COD concentration from PBS leaching, it lowered the leaching rate, which, as reported by Barkhordari et al. [28], would suppress microbial activity and thereby compromise nitrogen removal efficiency. The stabilized effluent concentrations of NH_4_^+^-N and NO_3_^−^-N also increased to 40.9 mg/L and 5.4 mg/L, respectively, accompanied by reduced removal efficiencies as low as 13.7% and 53.7%, respectively, which confirmed that both nitrification and denitrification processes were inhibited. Finally, the effluent TN concentration was as high as 47.7 mg/L, with a weakened removal efficiency of 20.8%. A reduction in temperature also significantly lowered pollutant removal efficiencies (*p* < 0.05, Figure S2), rendering effective nitrogen removal difficult at 5–7 °C.

In summary, temperature had the most significant impact on the NH_4_^+^-N removal efficiency, followed by the COD removal efficiency. When the temperature was above 23 °C, PBS could effectively enhance the nitrogen removal effect of low-carbon wastewater. However, when the temperature was below this level, there was a risk of effluent failing to meet the standards and thus not satisfying the WWTPs’ discharge requirements. 23–25 °C was recommended as a suitable operating temperature.

### 3.2. Decreasing Temperature Reduces Microbial Community Diversity

The performance of temperature-regulated wastewater treatment mainly depends on its impact on microbial community diversity. Key diversity indices at different temperatures were analyzed. As shown in Figure 2a, the Coverage indices at 32–34, 23–25, 14–16, and 5–7 °C were 0.99, 0.99, 0.99, and 0.99, respectively. This confirmed that the sequencing data were accurate and reliable, covering all microbial community information. The Sobs index is used to characterize community richness. The Sobs values of biosamples under these four temperature conditions were 782, 785, 763, and 723, respectively (Figure 2b). This indicated that the microbial community formed at 5–7 °C had lower observed species richness, which may be due to low-temperature stress selecting and enriching cold-adapted microbial groups [29]. The Shannon index is used to estimate microbial diversity in biological samples. A higher Shannon index indicates greater community diversity. The Shannon indices of biosamples under these four temperature conditions were 4.57, 4.63, 4.53, and 4.47, respectively (Figure 2c), suggesting that the community was mainly driven by the synergistic effect of various genera. In addition, the Shannoneven index reflects the evenness of species distribution in samples. For these four samples, the Shannoneven indices were 0.68, 0.68, 0.67, and 0.64, respectively (Figure 2d), which was consistent with the conclusion of Bai et al. [30] that low temperature reduces microbial community diversity. This further confirmed that at 5–7 °C, as species richness decreased, the microbial community exhibited lower diversity and a less uniform distribution of species abundance. Therefore, a gradual decrease in temperature may cause stress-induced community remodeling, reshaping the dominance structure of the microbial community. However, further evaluation is required to better understand the impact of temperature changes on differences in microbial communities.

### 3.3. Decreasing Temperature Drives the Microbial Community Reconstruction

How does decreasing temperature regulate microbial community reconstruction? At the phylum level, as shown in Figure 3a, at 32–34 °C, the dominant phyla were Proteobacteria (67.64%), Bacteroidetes (11.42%), Actinobacteria (10.48%), and Firmicutes (6.00%). Among them, Proteobacteria have been reported to exhibit efficient nitrogen removal performance [31], while Bacteroidetes, Firmicutes, and Actinobacteria have been confirmed to have the potential to degrade lignocellulose [32]. At 23–25 °C, the main dominant phyla were Proteobacteria (73.78%), Bacteroidetes (9.98%), Actinobacteria (8.11%), and Firmicutes (4.20%). The abundance of Proteobacteria and Actinobacteria increased, accompanied by a decrease in the abundance of other phyla, confirming that Proteobacteria and Actinobacteria were the key phyla contributing to pollutant removal performance. At 14–16 °C, Proteobacteria still dominated with a high abundance of 77.91%, yet nitrogen removal performance declined. This may be attributed to reduced microbial activity, which was also a key factor contributing to decreased nitrogen removal. Additionally, Bacteroidetes further decreased to 7.72%, resulting in a further reduction in COD removal efficiency. At 5–7 °C, the abundance of Proteobacteria dropped to 73.42%, along with reduced Firmicutes (3.64%) and Actinobacteria (3.77%), which further decreased pollutant removal efficiency and led to high effluent concentrations.

At the genus level, as shown in Figure 3b, the microbial community structure was analyzed to identify key genera. At 32–34 °C, *unclassified_Burkholderiaceae*, closely associated with nitrogen and COD removal [33], dominated with an abundance of 7.73%. *Leucobacter*, a heterotrophic genus reported to be thermophilic [34,35], also reached an abundance of 7.47%. *Hydrogenophaga*, a reported heterotrophic/mixotrophic denitrifying bacterium [36], also acted as a dominant denitrifying genus with an abundance of 6.22%. The secondary dominant genera *Flavobacterium* [37] (4.96%), *Dechloromonas* [38] (4.78%), *Zoogloea* (4.13%), *Allorhizobium-Neorhizobium-Pararhizobium-Rhizobium* [39] (3.65%), and *Desulfobulbus* [40] (3.31%) synergistically facilitated COD and nitrogen removal. At 23–25 °C, the abundances of *Hydrogenophaga*, *unclassified_Burkholderiaceae*, and *Desulfobulbus* continued to increase to 12.17%, 13.93%, and 5.06%, respectively, becoming the main dominant genera, and this explained the improved nitrogen removal performance. In contrast, the abundances of *Leucobacter*, *Flavobacterium*, and *Dechloromonas* decreased to 6.29%, 4.63%, and 4.58%, respectively, which was one of the factors contributing to changes in pollutant removal performance. At 14–16 °C, *unclassified_Burkholderiaceae, Hydrogenophaga*, and *Dechloromonas* still dominated with high abundances of 11.59%, 10.06%, and 5.10%, respectively. However, the abundances of *Leucobacter*, *Desulfobulbus*, and *Flavobacterium* gradually decreased to 2.82%, 3.93%, and 3.18%, respectively, leading to further declines in pollutant removal performance. At 5–7 °C, although *unclassified_Burkholderiaceae* (10.18%), *Hydrogenophaga* (9.15%), and *Desulfobulbus* (3.72%) remained dominant, their abundances showed a downward trend, confirming that these genera were more suitable for enrichment at higher temperatures. The abundance of *Leucobacter* decreased to 2.34%, which was also one of the reasons for the reduced COD removal efficiency. In contrast, the abundances of *Dechloromonas*, *Zoogloea* [41], and *Flavobacterium* increased to 5.56%, 4.73%, and 4.60%, respectively, indicating that these genera possessed low-temperature-adapted potential.

The co-occurrence network among genera was further explored (Figure 3c). The *unclassified_Burkholderiaceae* showed a significant positive correlation with *RBG-16-49-21*, *Hydrogenophaga*, and *Desulfobulbus*. The main metabolic pattern was that temperature changes drive dominant genera to achieve stable enrichment and pollutant removal through synergistic symbiosis. Additionally, *Dechloromonas* exhibited a significant positive correlation with *Rhodobacter*, *Phaeodactylibacter*, *norank_mle1-27*, *norank_Chitinophagaceae*, and *Thiothrix*. Secondary dominant genera also assisted in nitrogen removal *via* synergistic symbiosis.

In conclusion, decreasing temperature led to a succession from *Leucobacter*-dominated communities to those dominated by *Hydrogenophaga* and *unclassified_Burkholderiaceae*, while *Flavobacterium* and *Dechloromonas* exhibited low-temperature adaptability.

### 3.4. Decreasing Temperature Drives Differential Enrichment of Genes in Vital Metabolic Pathways

The predicted abundances of enriched functional pathways can reflect the metabolic potential of microorganisms, thereby requiring an investigation into their enrichment characteristics using the KEGG database. As shown in Figure 4a, at level 3, high temperatures (32–34 °C) promoted the enrichment of Carbon metabolism and Nitrogen metabolism, with abundances reaching 2.80‰ and 0.51‰, respectively, ultimately achieving the highest organic matter and nitrogen removal performance. Glycolysis/Gluconeogenesis is one of the key pathways regulating small-molecule metabolism, with an abundance as high as 0.87‰. It helps provide sufficient electron donors for heterotrophic microorganisms to carry out denitrification. The abundances of the two-component system and ABC transporters reached 2.64‰ and 2.35‰, respectively, indicating that microorganisms maintained high metabolic activity. At 23–25 °C, the abundances of Carbon metabolism and Glycolysis/Gluconeogenesis decreased to 2.72‰ and 0.80‰, respectively, leading to reduced organic matter removal performance. However, Nitrogen metabolism remained stable, ensuring consistent nitrogen removal performance, consistent with the results in Figure 1. At 14–16 °C, although the abundance of Nitrogen metabolism increased to 0.54‰, the abundances of Carbon metabolism and Glycolysis/Gluconeogenesis decreased to 2.71‰ and 0.83‰, respectively. Although the abundance of glycolysis/gluconeogenesis remained relatively high at 14–16 °C, the total abundance of carbon metabolism genes decreased, limiting electron donor supply. The abundances of Two-component system and ABC transporters increased to 2.72‰ and 2.57‰, indicating that the regulatory activity of microorganisms in response to environmental stress was enhanced to cope with the gradually decreasing temperature. At 5–7 °C, the decreased abundances of Carbon metabolism and Glycolysis/Gluconeogenesis still limited nitrogen removal performance. The Two-component system and ABC transporters further increased to 2.78‰ and 2.77‰, showing that the self-regulatory activity of microorganisms was further strengthened to withstand low-temperature stress.

Furthermore, the key pathways of carbon metabolism and nitrogen metabolism were predicted based on KEGG, along with their abundances (Figure 4b,c). The carbon metabolism process ultimately relies mainly on the glycolysis pathway to produce small-molecule organic matter as electron donors [42], which is used to enhance denitrification by heterotrophic denitrifying microorganisms. A total of 23 genes involved in carbon metabolism were analyzed. At 32–34, 23–25, 14–16, and 5–7 °C, the total abundances of these genes were 7.79‰, 7.35‰, 7.00‰, and 6.95‰, respectively. The glycolysis pathway was significantly affected by temperature, resulting in difficulty in providing sufficient electron donors at low temperatures, which was consistent with the conclusions at pathway level 3. Among these genes, *GAPDH*, *FBA*, and *gpmI* were the stably enriched dominant genes.

The abundance of genes involved in nitrogen metabolism was calculated. At 32–34, 23–25, 14–16, and 5–7 °C, the total abundances of nitrifying genes were 0.006‰, 0.010‰, 0.004‰, and 0.002‰, respectively. This indicated that low temperatures significantly inhibited the enrichment of nitrifying genes, which was also a key reason for the reduced nitrogen removal performance. Among denitrifying genes, although the proportion of *nirK* gene decreased from 0.017‰ at 32–34 °C to 0.011‰ at 5–7 °C, it still dominated and enabled denitrification. The *napA*, *napB*, and *nosZ* genes maintained relatively high abundances at low temperatures and were regarded as low-temperature-adapted genes, which was consistent with the conclusions in the studies by Dai et al. [43] and Tang et al. [44].

In a word, decreasing temperature mainly limits organic matter utilization efficiency by inhibiting the enrichment of carbon metabolism genes, thereby restricting the nitrogen removal process. It also limits the enrichment of nitrifying genes, ultimately reducing both nitrogen and COD removal performance.

### 3.5. Decreasing Temperature Restricts Molecular Mass Transfer and Limits Nitrogen Removal

Pollutant removal involves both biological and abiotic mechanisms. Although the temperature-driven gene enrichment characteristics have been analyzed through the above discussion, abiotic mechanisms remain underexplored.

Temperature-driven molecular mass transfer is an important factor limiting wastewater treatment. An amorphous unit cell was constructed based on the concentrations of four molecules (NH_4_^+^-N, H_2_O, C_6_H_12_O_6_, and NO_3_^−^-N) in the wastewater treatment system, and the diffusion coefficients of these four molecules were calculated. The corresponding molar quantity of C_6_H_12_O_6_ was calculated based on COD concentration, and the main nitrogen-containing molecules (NH_4_^+^-N and NO_3_^−^-N) were added in proportion. As an important substrate that provides electron donors, the trajectory of C_6_H_12_O_6_ molecules was visualized (Figure 5a), showing that 23–25 °C resulted in a larger diffusion range, while a decrease in temperature led to a slower mass transfer process. Further calculations confirmed that the diffusion coefficient of C_6_H_12_O_6_ molecules was the highest at 23–25 °C, reaching 0.22 Å^2^/ps (Figure 5b). At the same time, the highest diffusion coefficients of NH_4_^+^-N and NO_3_^−^-N were also achieved at this temperature, which were 1.23 Å^2^/ps and 0.66 Å^2^/ps, respectively. Efficient diffusion facilitates substrate transfer to microorganisms, thereby achieving higher organic matter utilization and nitrogen removal performance. Notably, the increasing mass transfer coefficient of H_2_O confirmed that higher temperatures improved mass transfer performance, while such a relationship did not hold for pollutants. In addition to temperature, concentration was identified as another key factor governing the mass transfer coefficient [45]. Accordingly, lower pollutant concentrations at high temperatures (32–34 °C) yielded smaller mass transfer coefficients compared with those at 23–25 °C. Additionally, below 14 °C, the diffusion coefficient was further reduced, which limited pollutant removal performance.

The molecular diffusion process is accompanied by efficient electron transfer. Three representative molecules, including NH_4_^+^-N, NO_3_^−^-N, and C_6_H_12_O_6_, were used to construct the amorphous cell. After excluding solvent effects, an implicit solvent system for electron transfer simulation was established. The charge transfer process between different atoms was analyzed by calculating the differential charge density. As shown in Figure 5c, from left to right across the unit cell, peaks represented charge accumulation, while valleys represented charge loss; a continuous decrease in temperature also led to smoother fluctuations. The repeated appearance of peaks and valleys confirmed the relatively high electron transfer activity in the system, which mainly involved the process of O gaining electrons and H losing charge (Figure 5d). Further calculations of the range of charge accumulation at different temperatures showed that the higher the temperature, the smaller the charge accumulation range, which was inconsistent with the pollutant removal performance observed in this study. This indicated that a lower pollutant concentration led to a smoother electron transfer process. Charge transfer level was not a key factor limiting pollutant removal.

In short, temperature drives molecular mass transfer to assist nitrogen removal, and high temperatures facilitate more efficient mass transfer than low temperatures. The excellent nitrogen removal performance at 23–25 °C is mainly attributed to stable metabolic activity and efficient mass-transfer processes, rendering this temperature range highly suitable for practical application.

### 3.6. Verification and Prediction of Vital Limiting Factors via Machine Learning

To further verify the temperature-driven biological and abiotic synergistic mechanism for nitrogen removal, a machine learning model based on a random forest was constructed. As shown in Figure 6a, the importance ranking of the diffusion coefficients of the four key molecules for achieving high COD removal efficiency and TN removal efficiency was consistent. Among them, the C_6_H_12_O_6_ diffusion coefficient was a universal key factor with the highest direct correlation to TN and COD removal efficiencies, which was in line with the research focus of this work. In addition, the importance of key genera was also calculated. The key genus affecting COD removal efficiency was *Dechloromonas*, followed by *Flavobacterium* and *Leucobacter* (Figure 6b). This is mainly because *Dechloromonas* and *Flavobacterium* exhibit low-temperature adaptability, which was consistent with the reports by Xu et al. [46] and Cong et al. [47], respectively, and they may play an important role in COD removal at low temperatures. The key genera affecting TN removal efficiency were *Leucobacter*, *Flavobacterium*, and *Dechloromonas* (Figure 6c). This further confirmed that a decrease in temperature led to a reduction in the abundance of the thermophilic genus *Leucobacter*, thereby lowering nitrogen removal efficiency. Targeted enhancement of these low-temperature-adapted genera *Flavobacterium* and *Dechloromonas* can significantly improve nitrogen removal performance.

Furthermore, the importance of functional genes predicted via the KEGG database was calculated. The *GAPDH*, *FBA*, and *gpmI* were important low-temperature-adapted genes limiting COD removal (Figure 6d), which was consistent with the results in Figure 4. In addition, *hao* was an important gene limiting TN removal, and the high NH_4_^+^-N concentration (caused by the limited role of *hao*) was a key factor leading to TN exceeding the standard, which aligned with the results in Figure 1. Moreover, the low-temperature-adapted denitrifying genes *nirK*, *napA*, and *napB* were also key genes assisting nitrogen removal (Figure 6e).

To provide optimization suggestions for practical applications, the model was trained using effluent NH_4_^+^-N concentration, effluent COD concentration, C_6_H_12_O_6_ diffusion coefficient, H_2_O diffusion coefficient, NH_4_^+^-N diffusion coefficient, and NO_3_^−^-N diffusion coefficient to predict effluent TN concentration. After training, the training set achieved an R^2^ of 0.996, an MSE of 3.34, and an RMSE of 1.80 (Figure 6f), and the test set achieved an R^2^ of 0.988, an MSE of 2.74, and an RMSE of 1.66 (Figure 6g). The high accuracy confirmed that effluent TN concentration could be predicted using the above parameters. Combined with SHapley Additive exPlanations for interpretation, it was confirmed that effluent NH_4_^+^-N concentration was closely correlated with effluent TN concentration (Figure 6h,i), followed by effluent COD concentration. It can be concluded that machine learning can predict key limiting factors based on wastewater composition, guide targeted reduction of these factors, and support the development of tailored treatment strategies.

In summary, machine learning based on the random forest model directly analyzes the complex relationships between different physicochemical parameters at both the macro and micro levels, verifies the key factors determining effluent TN concentration, and predicts treatment performance. This provides technical guidance for optimizing and designing efficient treatment strategies.

### 3.7. Application Implications

The synergistic regulation of wide-range temperature fluctuations on the coupled biotic-abiotic mechanisms of nitrogen removal systems has rarely been reported, limiting their guidance for variable-temperature operation in full-scale wastewater treatment plants. Across the 5–34 °C range, marked temperature changes coincided with successional shifts in nitrogen-removing microbial communities and associated nitrogen-cycling genes, while organic matter mass transfer efficiency fluctuated accordingly. Machine learning analysis further identified organic matter mass transfer efficiency as the primary limiting factor for nitrogen removal performance, with effluent NH_4_^+^-N concentration acting as the core constraint on final TN levels. Unlike earlier studies focusing merely on biological mechanisms, this work integrated microbial succession, functional gene potential, mass transfer characteristics and machine learning interpretation to illustrate the interactive “temperature + mass transfer efficiency + microbial community + nitrogen removal performance” linkage, improving the theoretical framework for PBS-based low-temperature nitrogen removal from low-carbon wastewater.

Experiments were carried out using synthetic wastewater without complex contaminants, which differs from real wastewater featuring fluctuating water quality and quantity and frequent low-temperature shocks. Further pilot-scale and field validation with real low-carbon wastewater is required to optimize process adaptability. Four key factors should be prioritized for real wastewater treatment at low temperatures: (1) Targeted enrichment of low-temperature-adapted core genera, including *Flavobacterium* and *Dechloromonas*, via microbial acclimation and biofilm immobilization to stabilize cold-adapted nitrogen removal communities. (2) Optimization of PBS carbon-release and mass transfer performance to mitigate low-temperature-induced diffusion limitation, match microbial metabolic rates and break mass transfer bottlenecks. (3) Machine learning-based water quality forecasting to precisely identify influent limiting pollutants such as NH_4_^+^-N, enabling targeted parameter regulation to prevent effluent nitrogen exceedance in advance. (4) Dynamic adjustment of HRT at low temperatures to balance treatment efficiency and operational cost. Future strategies combining machine learning prediction and self-adaptive temperature control can be developed based on the proposed mechanisms to achieve stable TN compliance at low temperatures.

For practical implementation, it is also necessary to calculate and reduce the costs associated with PBS consumption. PBS is a polyester polymer containing ester bonds susceptible to enzymatic hydrolysis by diverse microorganisms. These ester groups can be cleaved into water-soluble oligomers and monomers, which are ultimately mineralized to CO_2_ and H_2_O for microbial metabolism [48,49]. Compared with conventional soluble carbon sources (e.g., sodium acetate), PBS provides sustained carbon supply to improve nitrogen removal performance. Unlike liquid carbon sources (e.g., slurry, methanol), PBS avoids the risk of effluent COD overshoot caused by over-dosage and reduces subsequent post-treatment costs. According to previous results [7], approximately 100 g of PBS can sustain long-term operation for more than 200 days at a material cost of around 3 USD. By contrast, an equivalent COD dose of C_6_H_12_O_6_ (≈150 g) costs approximately 0.5 USD over the same 200-day period in lab-scale research. However, repeated dosing is required for soluble C_6_H_12_O_6_ carbon sources, leading to labour and maintenance costs far exceeding 3 USD and dominating total expenditure. Future modification strategies, solid waste-derived synthesis, and filler regeneration-reuse are expected to lower material costs and promote the practical application and economic feasibility of this PBS-based technology for low-carbon wastewater treatment.

Despite these insights, several methodological and data limitations should be acknowledged. The experiments were conducted at lab-scale using synthetic wastewater, which cannot fully represent real low-carbon wastewater with fluctuating loads and complex contaminants. The temperature range (5–34 °C) did not include rapid shocks or extreme conditions. Functional gene abundances were predicted from sequencing data, reflecting potential rather than actual metabolic activity. Molecular dynamics simulations used simplified amorphous cell models with an implicit solvent, which may not fully capture the heterogeneity of real biofilms. The random forest model was trained on a limited dataset from a single reactor configuration, restricting its generalizability. Moreover, the observed relationships among temperature, mass transfer, microbial succession, and nitrogen removal are mainly statistical associations rather than direct causal evidence. Therefore, the conclusions should be interpreted with caution, and further validation with real wastewater, multi-omics, and pilot-scale tests is needed.

## 4. Conclusions

This study investigated the differences in the treatment efficiency of PBS-enhanced low-carbon wastewater at temperatures ranging from 5 to 34 °C. It was found that when the operating temperature was in the range of 23–34 °C, nitrogen could be effectively removed, and the effluent met the discharge standards of WWTPs specified in China. However, when the temperature was lower than this range, there was a risk of the nitrogen concentration in the effluent exceeding the standard.

In terms of mechanisms, a large-scale decrease in temperature led to the succession from thermophilic microbial communities dominated by *Leucobacter* to those dominated by *unclassified_Burkholderiaceae* and *Hydrogenophaga*, as well as a decreased abundance of nitrifying genes and carbon metabolism genes, thereby inhibiting wastewater treatment performance. During this process, the efficient molecular diffusion process driven by high temperature was also a key factor regulating nitrogen removal. Machine learning verified a strong correlation between organic matter diffusion efficiency and nitrogen removal efficiency in low-carbon wastewater. *Leucobacter* was the dominant key genus at the relatively high temperature range of 32–34 °C, while *Flavobacterium* and *Dechloromonas* were potential key genera that functioned within the low temperature range of 5–25 °C. Results from the machine learning model also revealed that effluent NH_4_^+^-N concentration showed the strongest association with effluent TN concentration.

As an innovative contribution, this study emphasized that organic matter mass transfer efficiency is a potential key factor limiting nitrogen removal in low-carbon wastewater treatment, and also highlighted that its treatment schemes need to be designed based on the concentration of pollutant components. As an innovative implication, in wastewater treatment, machine learning technology should be combined with water quality prediction results to identify and control key limiting pollutant components in the influent, so as to improve the TN removal efficiency. These findings provide novel insights for the design and optimization of low-carbon wastewater treatment, especially for enhancement at low temperatures.

## Figures and Tables

**Figure 1:**
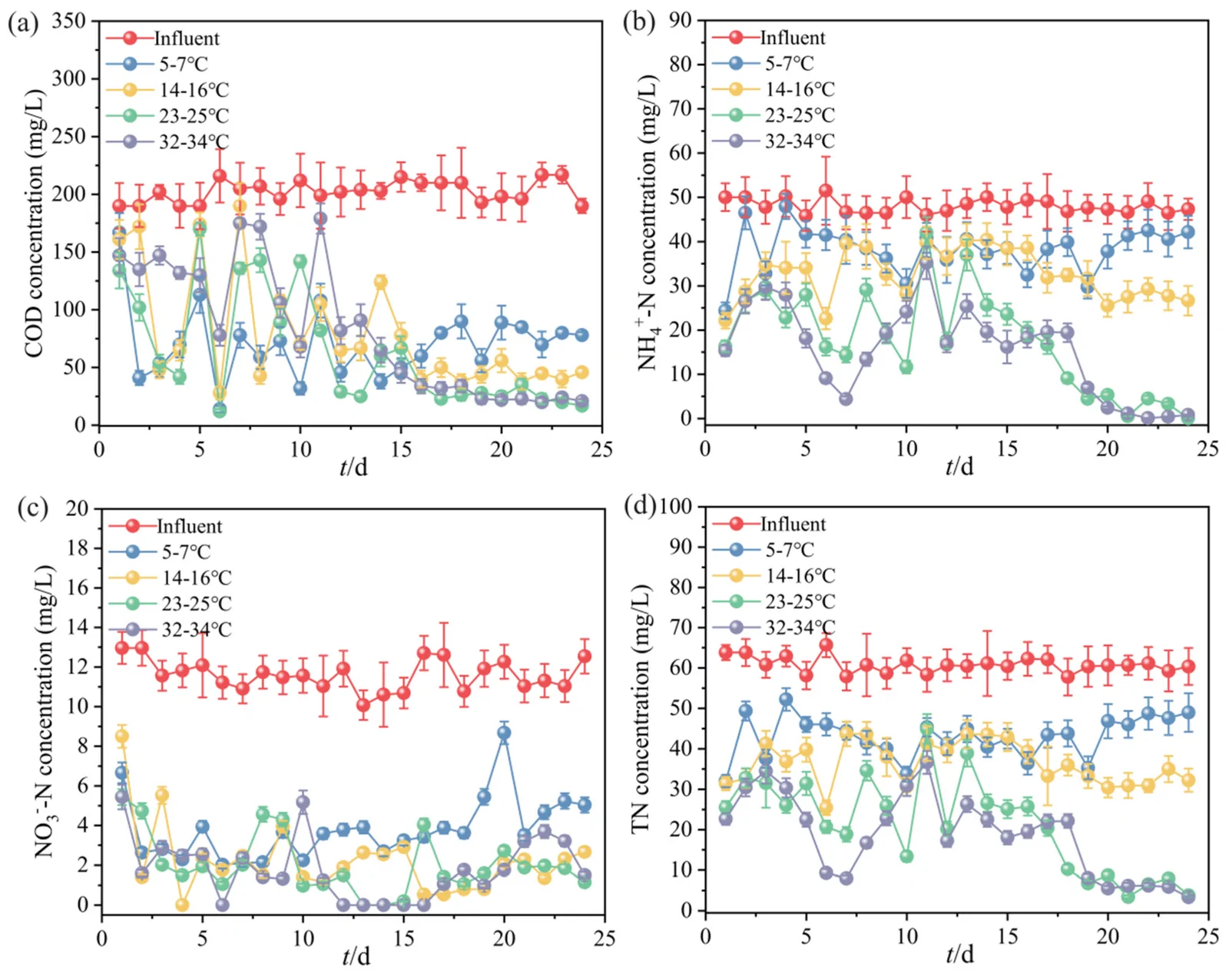
(**a**) COD removal performance, (**b**) NH_4_^+^-N removal performance, (**c**) NO_3_^−^-N removal performance, and (**d**) TN removal performance at different temperatures.

**Figure 2:**
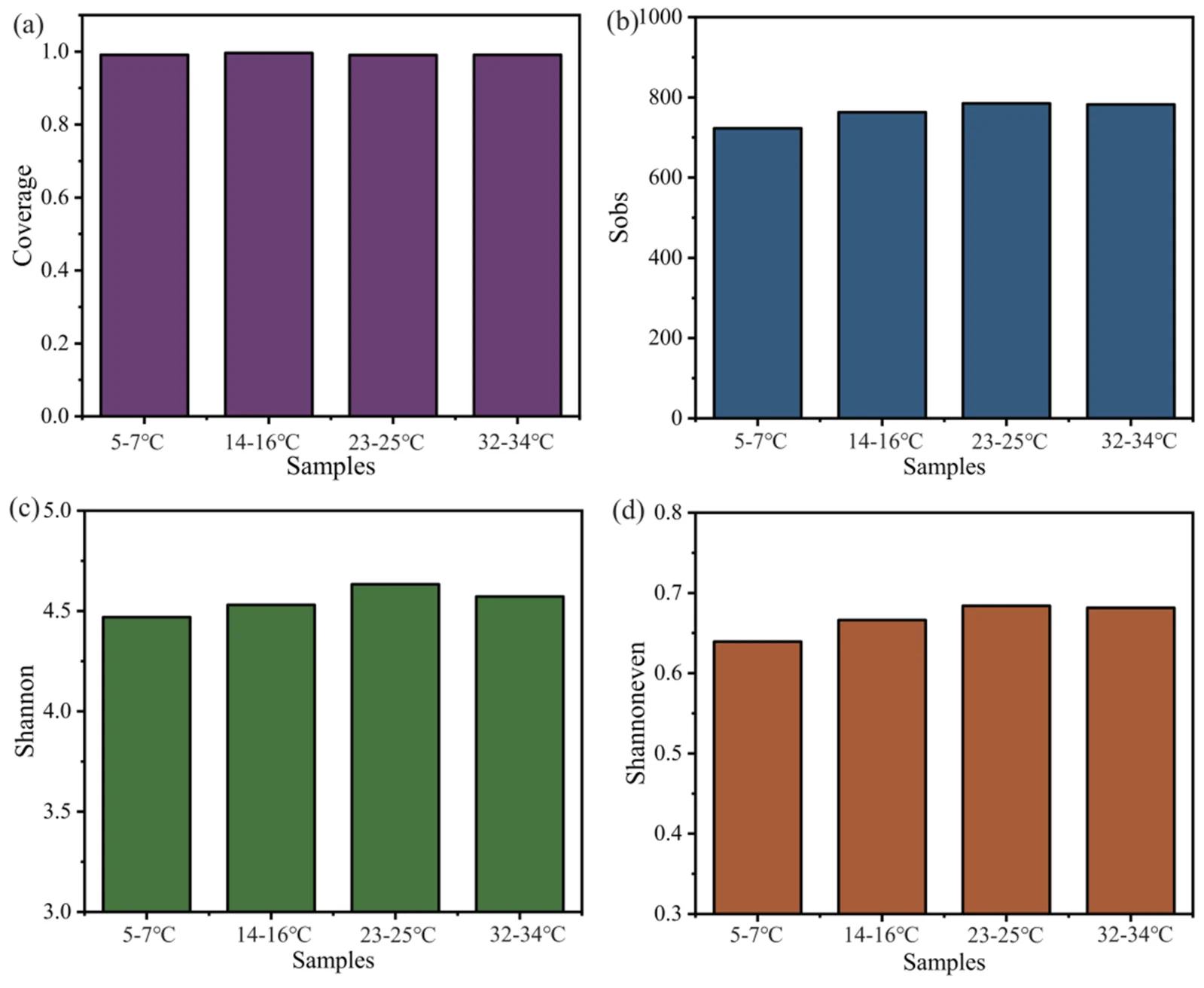
(**a**) Coverage, (**b**) Sobs, (**c**) Shannon, and (**d**) Shannoneven indices at different temperatures.

**Figure 3:**
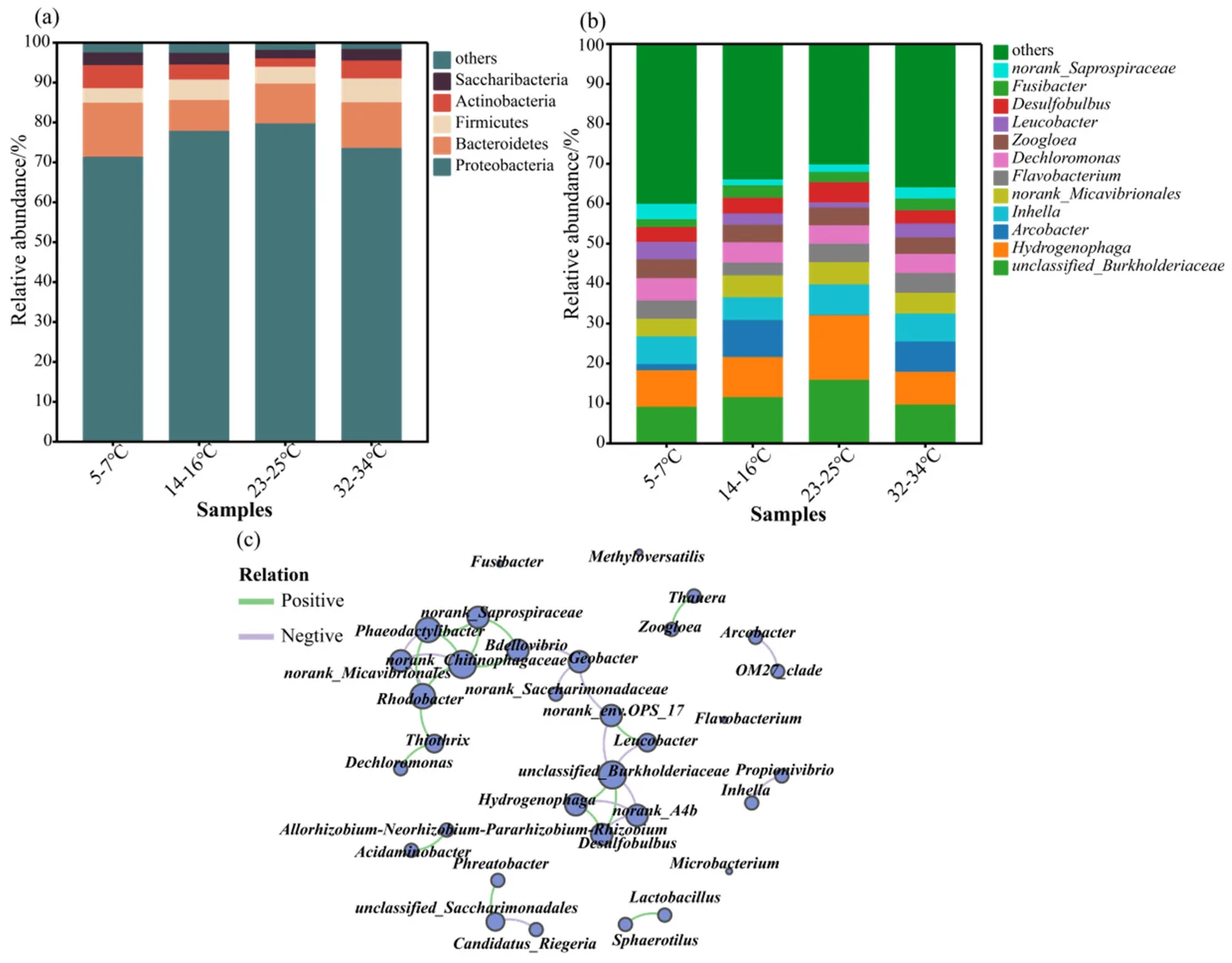
Community structure composition at the (**a**) phylum level and (**b**) genus level at different temperatures. (**c**) Co-occurrence network based on *Pearson* correlation (*p* < 0.05).

**Figure 4:**
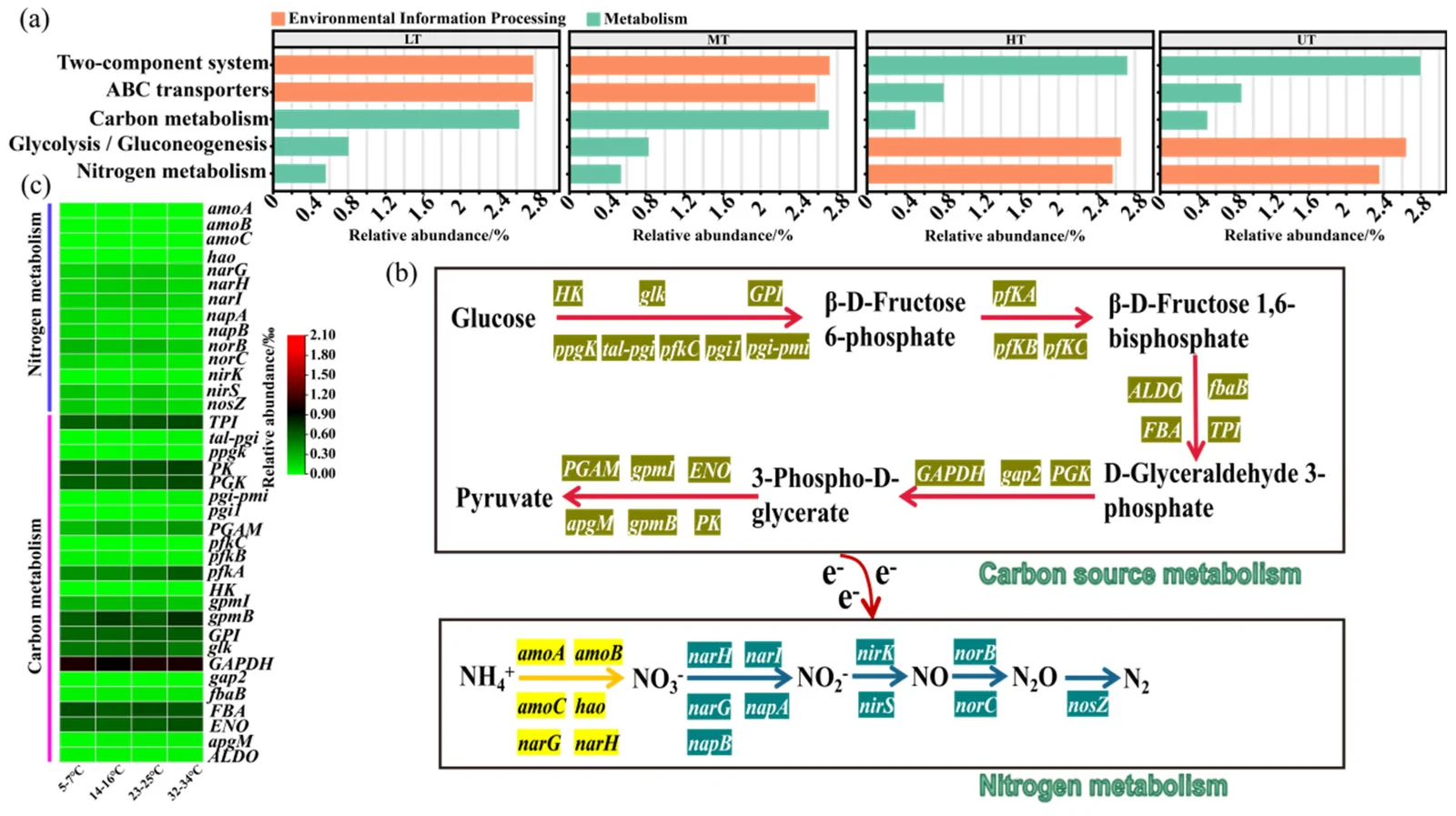
(**a**) Enrichment characteristics of functional pathways at level 3, (**b**) vital pathways involved in carbon and nitrogen metabolism, and (**c**) gene abundances of these pathways at different temperatures.

**Figure 5:**
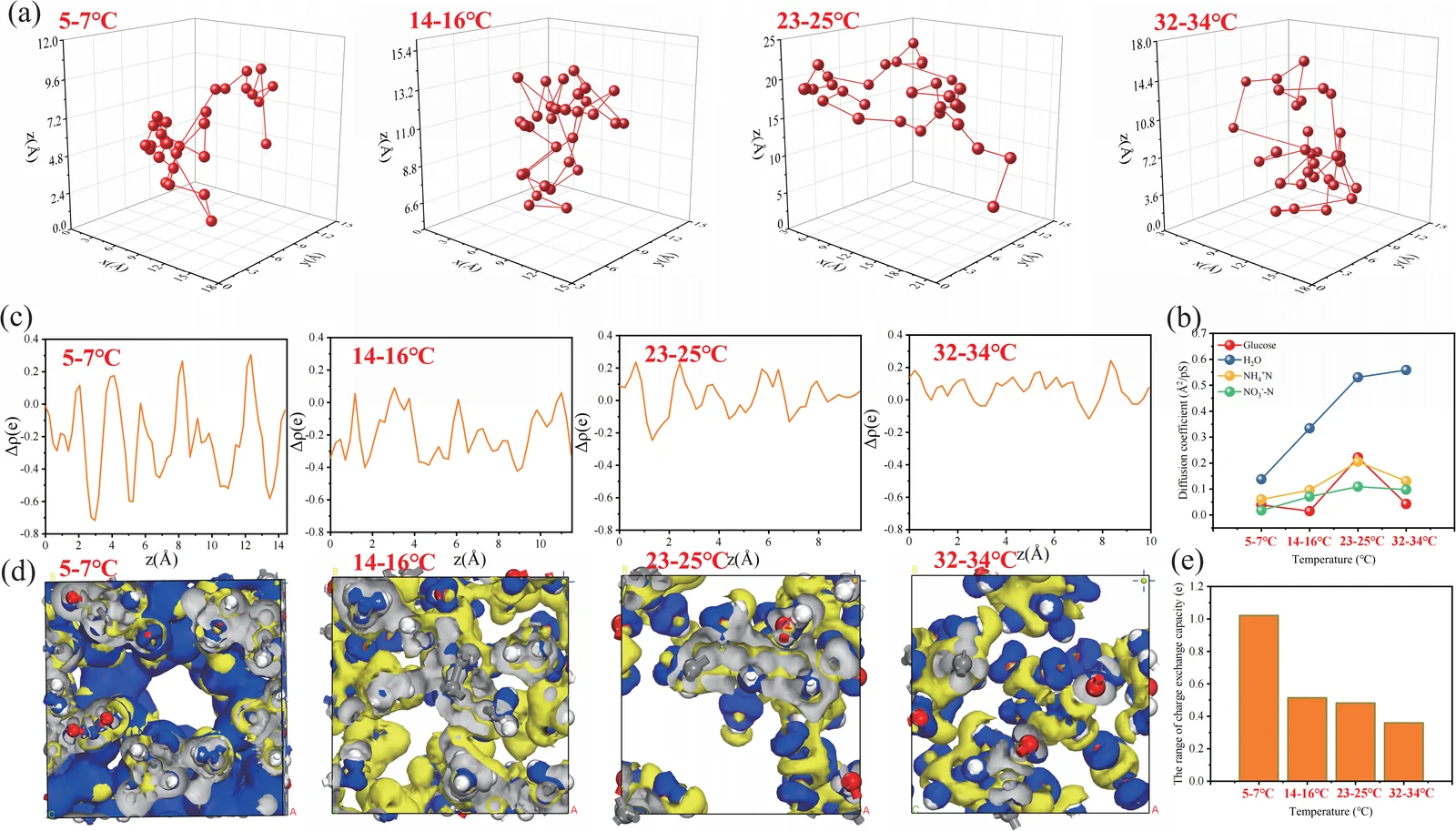
(**a**) Visualization of C_6_H_12_O_6_ mass transfer pathways, (**b**) diffusion coefficients of representative molecules, (**c**) differential charge accumulation trends, (**d**) their visualization, and (**e**) range of differential charge accumulation at different temperatures, where blue represents electron accumulation (electron gain), and yellow represents electron depletion (electron loss).

**Figure 6:**
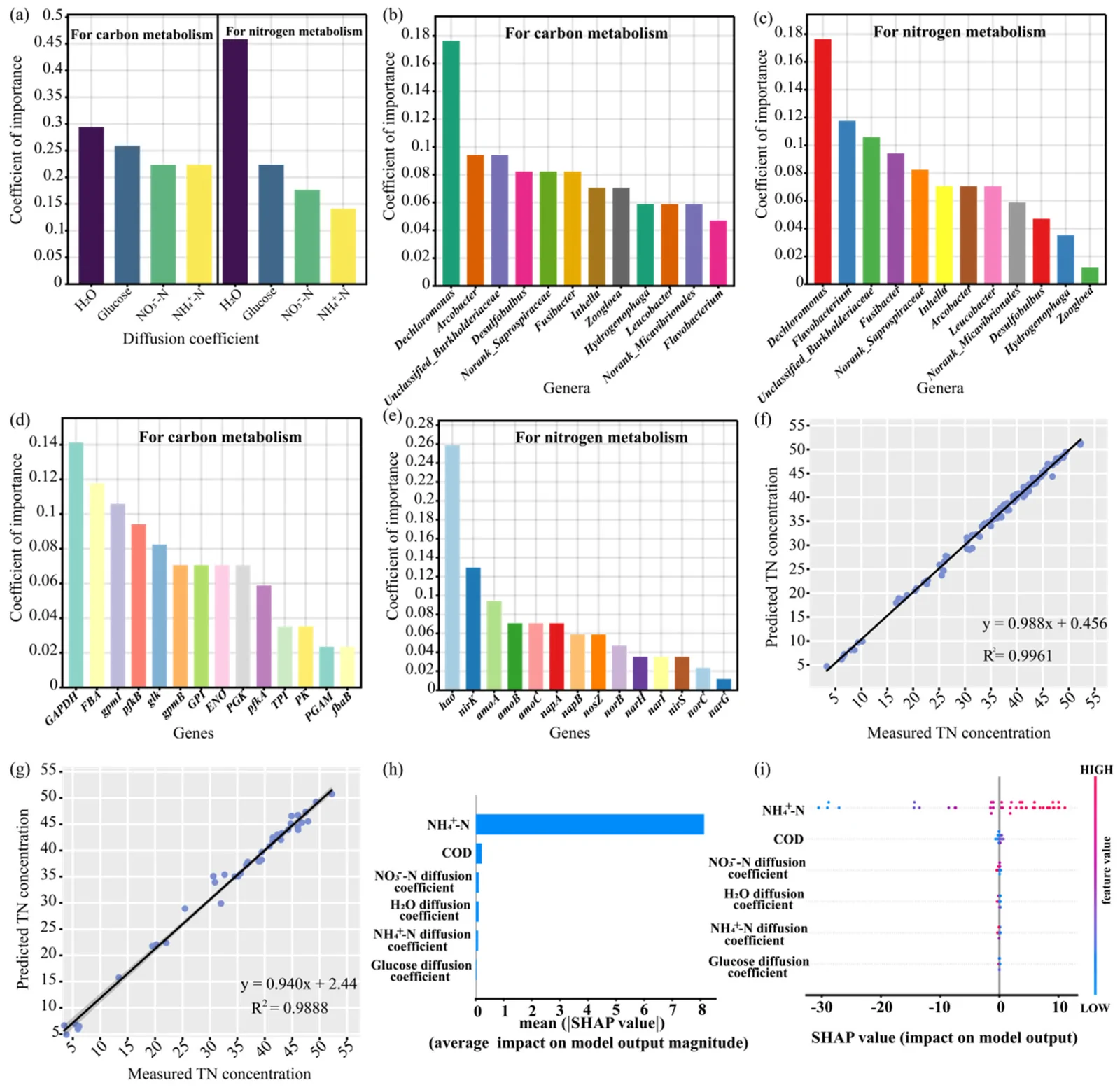
(**a**) Importance ranking of mass transfer coefficients. (**b**) Importance ranking of dominant genera for COD removal efficiency. (**c**) Importance ranking of dominant genera for TN removal efficiency. (**d**) Importance ranking of genes for COD removal efficiency. (**e**) Importance ranking of genes for TN removal efficiency. Prediction effect in the (**f**) training set and (**g**) test set. (**h**) Importance ranking of key factors affecting TN prediction, and (**i**) corresponding SHapley Additive exPlanations values.

## Data Availability

The original contributions presented in this study are included in the article/Supplementary Materials. Further inquiries can be directed to the corresponding authors.

## Supplementary Materials

The following supporting information can be downloaded at: https://www.mdpi.com/article/10.3390/microorganisms14102167/s1, Figure S1: Experimental setup diagram; Figure S2: Comparison of pollutant removal efficiency after stabilization under different temperature conditions.
