# Supplementary material for: Temperature Effects on Biotic and Abiotic Mechanisms of Nitrogen Removal Enhanced by Polybutylene Succinate in Synthetic Low-Carbon Wastewater
Source: Microorganisms. 2026 Sep 25;14(10):2167. doi: 10.3390/microorganisms14102167 (PMC13707421; doi:10.3390/microorganisms14102167)
Supplement: Supplementary file 1 [file microorganisms-14-02167-s001.zip › microorganisms-4498972-supplementary.pdf]

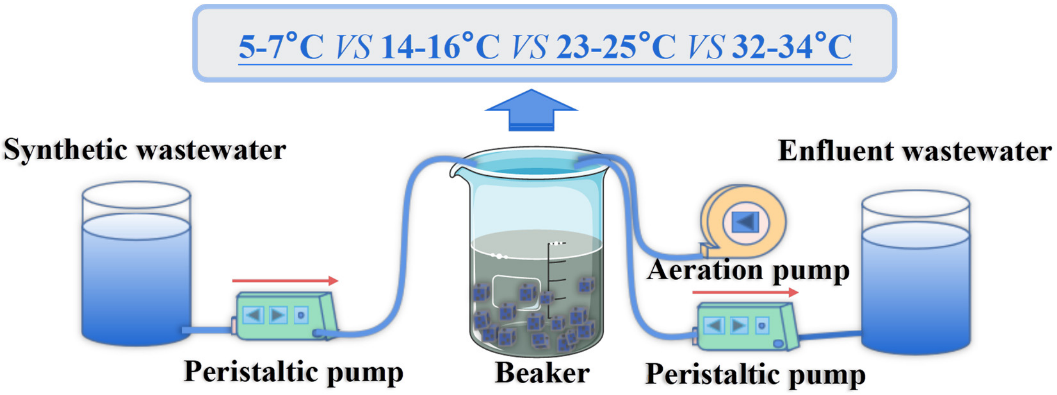

Figure S1. Experimental setup diagram

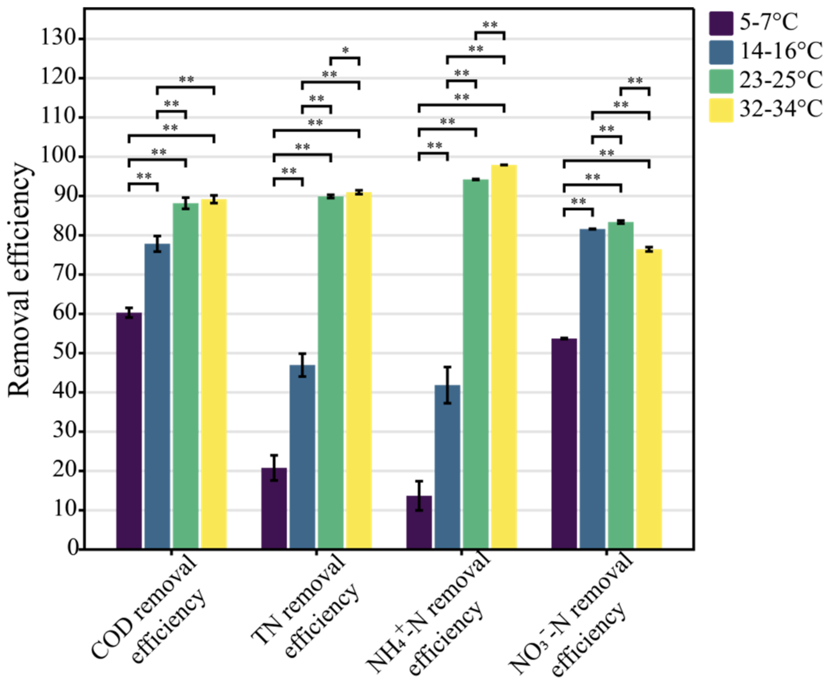

Figure S2. Comparison of pollutant removal efficiency after stabilization under different temperature conditions. *, $P < 0.05$; **, $P < 0.01$.
